# Special Protein Molecules Computational Identification

**DOI:** 10.3390/ijms19020536

**Published:** 2018-02-10

**Authors:** Quan Zou, Wenying He

**Affiliations:** School of Computer Science and Technology, Tianjin University, Tianjin 300354, China; hwying1234@tju.edu.cn

**Keywords:** bioinformatics, machine learning, feature selection, protein classification, network analysis, molecular docking

## Abstract

Computational identification of special protein molecules is a key issue in understanding protein function. It can guide molecular experiments and help to save costs. I assessed 18 papers published in the special issue of *Int. J. Mol. Sci.*, and also discussed the related works. The computational methods employed in this special issue focused on machine learning, network analysis, and molecular docking. New methods and new topics were also proposed. There were in addition several wet experiments, with proven results showing promise. I hope our special issue will help in protein molecules identification researches.

## 1. Introduction

With the development of next generation sequencing technologies, the size of biological databases have increased dramatically in terms of the number of samples. It is fast and cheap to obtain biological sequences but relatively slow and expensive to extract function information because of limitations of traditional biological experimental technologies. Protein, as the product of gene expression and the important material basis of life activity, participates in almost all life activities and biological processes. For some special protein molecules, the detection of new ones is time-consuming and costly. Some special proteins are present, such as cytokines, enzymes, cell-penetrating peptides, anticancer peptides, cancerlectins, and G protein-coupled receptors. In order to save the wet experimental costs, researches first select some candidates through computer programs. The “computer program” is the key step in selecting candidates. High false positive software would lead to high spending on the validation process.

In this special issue, these “computer program” approaches and algorithms are discussed. Numerous sequence-based “golden features” have been proposed for these problems, such as Chou’s PseAAC. Ever since the concept of PseAAC was proposed, it has penetrated into nearly all fields of protein identification. However, it is suggested that special features and classification methods should be proposed for special protein molecular. “Golden features” could hardly apply to all kinds of proteins. In this special issue, submissions focused on a kind of special protein molecules, collected related data sets, got better prediction performance (especially low false positive), and developed friendly software tools or web servers.

**Figure 1:**
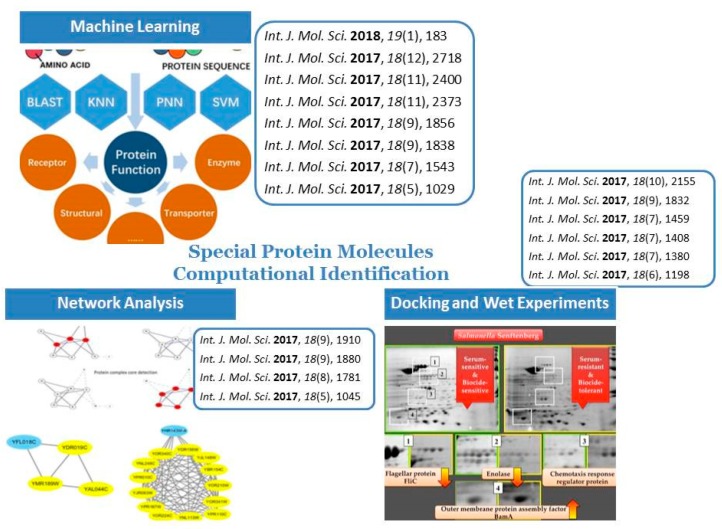
Subtopics of our special issue [38,52,63].

We received 36 submissions. After rigorous reviewing process, 18 papers were published. They come from different countries, including China, Russia, Canada, Australia, USA, Poland, etc. These papers could be categorized into three subtopics. As shown in Figure 1.

## 2. Machine Learning Related Researches

### 2.1. Protein–Protein Interaction Prediction

The first subtopic is to identify or predict protein function with machine learning methods. Two papers focused on protein–protein interaction prediction. Protein–protein interactions (PPIs) play crucial roles in almost all cellular processes. Correctly predicting protein–protein interactions contributes to precise protein function prediction [1,2]. Most of them focus on the PPIs predictions from various data types, including 3D structural information, gene ontology and annotations, and gene fusion. Wang et al. [3] proposed a sequence-based approach (DNN-LCTD) combining deep neural networks (DNN) and Local Conjoint Triad Description (LCTD) feature representation. Experimental results showed that DNN-LCTD is very promising for predicting PPIs. Wang et al. [4] using the Zernike moments (ZM) descriptor on the PSSM combined with Probabilistic Classification Vector Machines (PCVM) classifier developed the PCVMZM predictor for predicting the PPIs from protein amino acids sequences. It was proved to be a robust, powerful and feasible PPI prediction method. Ding et al. [5] developed a random forest algorithm based predictor using a multivariate mutual information feature representation scheme and normalized Moreau-Broto Autocorrelation information from protein sequence. Another work [6] is a novel matrix-based protein sequence representation approach to identify PPIs, using an ensemble learning method for classification. The matrix of Amino Acid Contact (AAC) was constructed based on the statistical analysis of residue-pairing frequencies in a data-set of 6323 protein–protein complexes. The feature vector was extracted by applying algorithms of Histogram of Oriented Gradient (HOG) and Singular Value Decomposition (SVD) on the Substitution Matrix Representation (SMR) matrix of protein sequence.

Drug-target interaction is a special PPI. Because the experimental prediction of drug-target interaction (DTIs) is time-consuming and expensive, computational technology with high accuracy plays a crucial rule in the large-scale rapid prediction of DTIs. Shen et al. [7] proposed DAWN a kind of Drug-target interactions predictor combining discrete Wavelet transform and Network features. Most importantly, DAWN as a kind of machine learning approach of feature vector-based method, has the desired effect under the condition of without network information. In the same year, they also developed the second tool [8] using molecular substructure fingerprints, Multivariate Mutual Information (MMI) of proteins, and network topology. 

Hotspot has important significance in the determination of protein–protein interactions [9]. Many methods have been developed for the hotspot predictions [10,11] and even protein binding site predictions [12]. Most of the works focused on the hotspot predictions from a curated small partial dataset of the whole protein sequences [13]. In Jiang’s work [14], the issue of hotspot determination was approached from whole natural protein sequences, and a random projection ensemble system based on k nearest neighbor algorithm to identify hotspot residues by sequence information alone was developed. Experimental results showed that although this method did not perform well enough in the real applications of hotspots, it was very promising in the determination of hotspot residues from whole sequences.

### 2.2. Special Proteins Identification

Besides protein–protein interaction, DNA binding proteins, ion channel proteins, and amyloids have also attracted researchers’ attentions. DNA binding protein is a kind of special protein molecule, whose identification is one of the most important tasks in studying the function of proteins. In this regard, many computational predictors have been proposed [15,16,17,18,19,20,21]. In a special issue, Zhang et al. [22] proposed a new approach to extract evolutionary information from the Position Specific Frequency Matrix (PSFM) and incorporate the evolutionary information, and a computational predictor was proposed for DNA binding protein identification. Experimental results showed that this predictor outperformed some existing state-of-the-art approaches in this field. DNA-protein interactions play a key role in a variety of biological processes, especially in cellular metabolism. Endowed with a ditto multi-scale idea in essence, Shen et al. [23] addressed a kind of competitive method called Multi-scale Local Average Blocks (MLAB) algorithm. Different from the structure-based route, MLAB exploited a strategy that not only extracted local evolutionary information from primary sequence, but also used predicted solvent accessibility. Moreover, the construction on the predictor of DNA-protein binding sites wields an ensemble weighted sparse representation model with random under-sampling. 

Ion channels are membrane proteins which are widely distributed in all cells. They have been shown to be extensively involved in various physiological and pathological processes, including regulating neuronal and cardiac excitability, muscle contraction, hormone secretion, fluid movement, and immune cell activation. Different ion channels play their unique roles in different biological processes. With the rapid development of next-generation sequencing technologies, the accumulation of proteomic data provides uswith a platform to systematically investigate and predict ion channels and their types. Several studies have focused on the prediction of ion channels and their types [24,25,26]. The paper published in the special issue [27] proposed a new prediction model to quickly predict ion channels and their types. An improved feature extraction method combining dipeptide composition with the physicochemical property correlation between two residues was developed to formulate protein samples. Subsequently, the analysis of variance (ANOVA) combined with the incremental feature selection (IFS) was employed to find out the optimal features which can produce the maximum accuracy. As a result, authors achieved the overall accuracies of 87.8% for discriminating ion channels from non-ion channels, 94.0% for distinguishing between voltage-gated ion channel and ligand-gated ion channels and 92.6% for four types of voltage-gated ion channels, respectively. Based on the proposed models, a web server called IonchanPred 2.0 (http://lin.uestc.edu.cn/server/IonchanPredv2.0) was established. The free predictor will be most useful to most wet-experimental scholars. A few groups have focused on the outer membrane protein recently, Wang et al. introduced the predicted topology structure as a mainly structure-specific feature to this classical type of ion channel protein, improved the precision of outer membrane identification [28], inter-barrel contact prediction [29] and fold recognition [30].

In this special issue, Antonets et al. [31] detected amyloidogenic proteins in the proteomes of plants. Amyloids are protein fibrils with characteristic spatial structure. The main computational method for them was phylogenetic analysis together with machine learning techniques. This kind of protein also includes DNA and RNA binding ones, which showed that different kinds of proteins have comment characters. To summarize, effective protein features and machine learning techniques are still essential and challenging in the future.

### 2.3. Protein Subcellular Localization and Function Analysis

Besides PPI, special proteins, protein subcellular localization, and function prediction are traditional challenges and attract researchers. In general, only when the protein is located in the correct subcellular location, can the protein function normally. Therefore, prediction of protein subcellular localization is an important component of proteomics, and it can aid the identification of drug targets. Due to the technical limitation and high cost of time and money in traditional experimental methods, research on protein subcellular location annotation with the machine learning technique has become a focused research problem in bioinformatics. When we use machine learning technologies to predict protein subcellular location, we need to extract the features of protein sequences, and then use the classifier to realize the protein classification. Thus feature extraction and dimension reduction are important techniques for analyzing the complex and high dimensional biological data in protein subcellular location. In order to improve the prediction accuracy of protein subcellular location, an appropriate algorithm for reducing data dimension should be used before classification. Wang et al. [32] proposed two feature fusion expressions and then used the linear discriminant analysis (LDA) method for dimension reduction. Considering the general nonlinear property in protein sequence data, they [33] introduced the nonlinear kernel discriminant analysis (KDA) method to reduce the high dimensionality in some feature data in this special issue. In this paper, an improved Gauss kernel parameter selection algorithm was proposed to predict subcellular location. It was proposed by maximizing the differences of reconstruction errors between edge normal samples and internal normal samples. The proposed method did not only show the same effect as traditional methods, but also reduced the computational time and improved the efficiency. It should be noted that LDA and KDA methods cannot only reduce the data dimensionality, but also take use of some classification information in the data, resulting in an ideal classification effect. Besides, there have been some new dimensional reduction algorithms which have been tried in other pattern recognition fields, such as face recognition [34]. 

Knowledge of protein function is the key to the understanding of the biological process and disease development and to the discovery of new therapeutic targets [35]. Various in-silico methods have been developed for protein function prediction [36], which complement one another due to their distinct underlying theory [37]. A comprehensive comparison of the performances between those popular prediction algorithms was conducted based on the information from 93 functional protein families [38], which observed a substantially higher sensitivity of BLAST and a significantly reduced false discovery rate of machine learning.

Since machine learning is a key issue in protein research, it is essential to extract numerical features from the protein primary sequence. Some recent studies showed that evolutionary information and the sequence-order effects are very important for extracting the features of proteins [39,40]. In their special issue, Du et al. [41] developed the UltraPse program to convert biological sequences into digital features. Unlike the PseAAC-Builder [42] or PseAAC-General [43], the UltraPse program can be used on DNA/RNA sequences as well as protein sequences. The program is a good starting point in predicting special protein functional characters, especially the exact subcellular localization of proteins [44].

## 3. Network Techniques Related Researches

Network analysis is also an important technique for protein identification and function research. Identification of disease genes is very important in medicine. For a disease, extracting its disease genes as completely as possible is helpful in understanding its pathogenesis, thereby designing effective treatments. To date, several network methods have been proposed to identify genes related to different diseases, such as the guilt by association (GBA) based method [45], the shortest path algorithm based method [46,47,48], the flow propagation algorithm [49], and the random walk with restart (RWR) algorithm based method [50]. In view of the fact that the RWR algorithm can make full use of the whole network, a RWR algorithm based method was proposed by Lu et al. [51] to identify disease genes of uveitis, a serious eye disease that may cause blindness in both young and middle-aged people. The method first applied the RWR algorithm on a protein–protein interaction (PPI) network using validated uveitis-related genes as seed nodes. Second, the obtained genes were filtered by a permutation test that can exclude false positive genes produced by the PPI network. Finally, they extracted important genes from the remaining genes by evaluating their associations to validate genes. Several putative genes were accessed and some have been determined to be important for the pathogenesis of uveitis.

Li et al. [52] employed the advanced network clustering algorithm for protein complex identification. Their method could detect the overlapping complex from the PPI network. Cluster analysis of biological networks is an important topic in systems biology. Up to now, a number of computational methods and tools have been proposed for analyzing biological networks and identifying protein complexes [53]. Various plugins based on cytoscape, such as CytoNCA [54], ClusterViz [55], DyNetViewer [56], CytoCtrlAnalyser [57], were developed to analyze biological networks from different perspectives. CytoCluster [58] in our special issue is a popular clustering tool which integrates six clustering algorithms and BinGO function. Since it was established in July 2013, CytoCluster has been downloaded more than 11,200 times from the Cytoscape App Store and has been applied to different biological networks analyses.

## 4. Docking and Wet Experiments Researches

Docking is still an interesting and hot topic in protein structure and function analysis, especially in the drug design process. Adenosine monophosphate-activated protein kinase (AMPK) plays a critical role in the regulation of energy metabolism. Huang et al. [59] employed molecular docking to get potential β1-selective AMPK activators. Finally, 12 novel compounds were selected as potential starting points for the design of direct β1-selective AMPK activators. Hou et al. [60] investigated the relationship between scopoletin structure and *TcPMCA1*(a gene name)-inhibiting activity of scopoletin and other 30 coumarin derivatives by employing docking and three-dimensional quantitative structure-activity relationships (3D-QSAR). This work offers additional insights into the mechanism underlying the interaction of scopoletin with *TcPMCA1* gene. Together with this work, the other three works in this special issue also carried out wet experiments. Besides wet experiments, Ding et al. [61] completed bioinformatics analysis and molecular dynamics simulation on glucose 1-dehydrogenase (GDH). Chandler et al. [62] extracted insulin-binding protein and insulin-like peptides in the Eastern spiny lobster, *Sagmariasus verreauxi*. Molecular modelling, including docking, showed various interaction and regulation. Futoma-Koloch et al. [63] laid special stress on analyzing the relationship between triamine-biocide tolerance of *Salmonella enterica* serovar Senftenberg with antimicrobial susceptibility, serum resistance, and outer membrane proteins.

To conclude, papers in this special issue cover several emerging topics of computational identification and bioinformatics analysis of special protein molecules. We fervently hope that this particular issue will attract considerable interest in the relevant fields. We are grateful to *Int. J. Mol. Sci.* for providing the chance to organize this special issue. We also thank the reviewers for their efforts in guaranteeing the high quality of this special issue. Finally, we thank all those who contributed to this special issue. *Int. J. Mol. Sci.* has promised to continue with the same topic as a new special issue in 2018. Besides special protein molecules, nucleic acids with special modifications identification (such as RNA m6A [64], protein phosphorylation [65] and methylation, etc.) will also be welcomed in the 2018 special issue. I hope more authors and readers will contribute, especially to the follow-up works from this special issue.
